# Current State of Type 1 Diabetes Immunotherapy: Incremental Advances, Huge Leaps, or More of the Same?

**DOI:** 10.1155/2011/432016

**Published:** 2011-07-18

**Authors:** Brett Phillips, Massimo Trucco, Nick Giannoukakis

**Affiliations:** ^1^Division of Immunogenetics, Department of Pediatrics, Children's Hospital of Pittsburgh, University of Pittsburgh School of Medicine, 4401 Penn Avenue, Pittsburgh, PA 15224, USA; ^2^Department of Pathology, Children's Hospital of Pittsburgh, University of Pittsburgh School of Medicine, 4401 Penn Avenue, Pittsburgh, PA 15224, USA

## Abstract

Thus far, none of the preclinically successful and promising immunomodulatory agents for type 1 diabetes mellitus (T1DM) has conferred stable, long-term insulin independence to diabetic patients. The majority of these immunomodulators are humanised antibodies that target immune cells or cytokines. These as well as fusion proteins and inhibitor proteins all share varying adverse event occurrence and severity. Other approaches have included intact putative autoantigens or autoantigen peptides. Considerable logistical outlays have been deployed to develop and to translate humanised antibodies targeting immune cells, cytokines, and cytokine receptors to the clinic. Very recent phase III trials with the leading agent, a humanised anti-CD3 antibody, call into question whether further development of these biologics represents a step forward or more of the same. Combination therapies of one or more of these humanised antibodies are also being considered, and they face identical, if not more serious, impediments and safety issues. This paper will highlight the preclinical successes and the excitement generated by phase II trials while offering alternative possibilities and new translational avenues that can be explored given the very recent disappointment in leading agents in more advanced clinical trials.

## 1. Introduction

Type 1 diabetes is an autoimmune disease clinically characterized by hyperglycemia underlai by a significant loss of pancreatic insulin-producing beta cell mass. Even though normoglycemia is achieved with pharmacologic insulin replacement, the underlying autoimmune response that impairs and eventually eradicates the beta cells is not treated. Insulin replacement cannot prevent the peripheral complications, a major source of patient morbidity and mortality. Strategies like beta cell replacement with cadaver donor islets still face the impediment of autoimmunity in addition to allogeneic rejection. There is therefore a need to develop methods that directly suppress or eliminate autoimmunity and allow a possible regenerative process. 

Activated autoreactive T cells are the mediator of beta cell destruction and therefore a prime therapeutic target. Other T cell subpopulations help determine the responsiveness of cytotoxic T-cells. T helper (Th) cells are one of these populations and are divided into 3 groups based on their cytokine production profiles: proinflammatory Th1 and Th17 and anti-inflammatory Th2. The balance of Th cell populations is an important regulator of the immune system and is often examined after immunotherapy treatments, along with anti-inflammatory T-regulatory (Treg) cells. In addition to these cell types, antigen-presenting cells (APCs) such as dendritic cells (DCs) and B cells are responsible for the direct activation of T cells in response to specific antigens. Various techniques of immunomodulation have been employed in animal models to directly or indirectly regulate cytotoxic T-cell activation utilizing these different target cell populations. Here we will discuss their progress through clinical trials and offer some commentary on whether they represent incremental advances, huge leaps in terms of curative outcome and/or improvement of insulin requirements, or more of the same.

## 2. To Prevent or to Reverse?

The identification of multiple genetic susceptibility loci over the past decade, when coupled with the presence in high titers of the traditional autoantibody markers in first-degree relatives of T1DM patients, offers a preventive interventional opportunity. By initiating immunomodulation in such pre-clinically diabetic individuals, it is theoretically possible to mitigate clinical onset of the disease. Statistically, a variety of modeling outcomes suggest that such an approach could be beneficial, although much of the optimism rests on biological data from mouse studies which may not be mirrored in humans. Furthermore, even though genetic and humoral risk may be considerable, they do not always result in clinical disease [75]. The therapist thus faces two dilemmas: (i) are the benefits of prevention worth the risks of the adverse events of current immunomodulation approaches? and (ii) are the benefits of prevention worth the considerable logistical outlays required to screen and treat all those who meet “high-risk” status? The first is the most germane, especially since the long-term effects on the immune system of newer immunomodulation agents are unknown. Furthermore, there are the real risks that latent infections due to dormant viruses could become productive and life threatening as well as the possibility that modulation of immune cells could provoke latent or low-grade autoimmunity other than T1DM. These valid arguments form the cornerstone against which any preventive immunomodulation approach will have to push to successfully enter clinical trials other than phase I safety studies.

On the other hand, attempting immunomodulation in individuals who exhibit clinical disease is better justifiable as the autoimmunity is not speculative (unlike in prevention approaches) but a fact. This then leads to the question of what is considered the point of “too late” at which immunomodulation is ineffective and only adverse events will plague the patient without any possibility of real benefit.

The most straightforward answer is to identify a time window that defines a period between the onset of clinical disease and the last possible point inside which immunomodulation will result in the preservation and/or restoration of a beta cell mass adequate enough to reduce the concentration of, or even obviate, exogenous insulin replacement. Traditionally, this window has been termed the “honeymoon” period; however, a number of studies suggest that it can extend further on, as C-peptide can be detected in adult individuals who have the disease for many years [76, 77]. The diabetic inflammation of the islets of Langerhans need not be associated with beta cell destruction. It is now well accepted that insulitis impairs beta cell sensing of glucose with/without concomitant impairment in insulin production and secretion in the absence of significant irreversible physical elimination of beta cells [78–80]. Studies also show that beta cells can be differentiated from pancreatic progenitors when inflammation is controlled and suppressed. In fact, under some conditions, suppression of islet inflammation also can be permissive for the replication, even if limited, of existing beta cells [81–84]. The key then is to find a means of first suppressing the islet inflammation to restore the function of residual beta cells and then, once this is secured, to identify a method to ensure that beta-cell-reactive T cells are prevented from reaching the islets and/or that their function is silenced. This second requirement necessitates the establishment of some form of tolerance or regulation of autoreactive immune cells. A product that achieves both concurrently would be best. 

## 3. Biomarkers of Efficacy

Although a number of surrogate measurements are accepted in clinical trials, thus far there are no bona fide biomarkers of therapeutic success. The presence of autoantibodies supports the autoimmune nature of T1DM and differentiates it from type 2 diabetes and other forms of metabolic glucohomeostasis impairment, but their disappearance cannot yield information on success of therapies. Cellular proliferation to islet-specific proteins and peptides can differentiate normal from disease-susceptible individuals; however, no causal relationship between disease process and specific cellular response has been established. Frequencies of cell populations in peripheral blood associated with immune regulation (i.e., Foxp3+ Tregs) have been suggested as markers of disease severity and stage and success of immunomodulation, but there is very little functional evidence in support of these propositions. Thus, in the absence of specific and therapy-related biomarkers, the field has accepted physiologic end-points as surrogates of therapy efficacy. 

In the absence of bona fide biomarkers of immune efficacy, physiologic measurements become of paramount importance. Examples, for T1DM, include maintenance or increase of pretreatment C-peptide levels, improvement of mixed meal-stimulated insulin production and consequent reduction of glucose levels in blood, improvements in glycated HbA1c and, in certain instances, reduction of exogenously administered insulin or complete cessation thereof for a period of time. As will be discussed later, the values between pretreatment and posttreatment physiological measurements in a number of recent immunomodulation clinical trials, in particular if promoted and implemented close to the clinical onset of the disease, have required stringent statistical analysis to identify differences, and in many respects, the pronouncements of success for some immunomodulation treatments require considerable faith in the outcome of statistical treatments of the data.

The frustration with many immunomodulation trials is the disconnect between preclinical data in mouse and rat models of T1DM and the physiologic outcomes in T1DM patients. 

## 4. Preclinical Models: Is Efficacy Predictive of Outcome in Humans?

Currently, there are only two spontaneously occurring, genetically susceptible animal models of T1DM: the heavily popular nonobese diabetic/LtJ strain (NOD) and the diabetes-prone biobreeding rat (BB) [85]. The intensity with which many studies are performed in these two strains overlooks some obvious, for the sake of clinical translation, inconvenient facts. First, the two strains are analogous to clones of two individual humans. Second, the differences between man and murine (or rat) immune systems, responses, and immunopathology of T1DM is as different as are the known and perceived similarities [85]. The lymphopenia that characterises the BB rat model is counterintuitive to how autoimmunity develops in this species, even though the target of immune dysfunction and destruction are the islets. Further confounding the issue is the presence of other autoimmunities and inflammatory conditions in the NOD mouse, calling into question just how tissue restricted is its autoimmunity. Another difference lies in the difference in the incidence and prevalence of the disease between female and male NOD mice, a difference that is not observed in humans. The predictable time-at-onset of disease in NOD mice and BB rats is not as simple to extrapolate to humans. Interindividual heterogeneity in humans lies in the different HLA susceptibility alleles, the non-HLA susceptibility loci, the primary autoantigen(s), and the actual composition of peripheral tolerogenic cell populations that wax and wane during preclinical disease progression. Even at the time of clinical onset, the actual mass of functional and potentially functional residual beta cells is unknown, and this heterogeneity can determine success or failure of a particular immunomodulator. 

What is sobering is that, of more than 230 therapeutic strategies employed (to various levels of success) in NOD mice and BB rats, fewer than ten have proven to be of any clinical utility, and therefore, only a few have progressed to advanced trials [85, 86]. This should caution the investigator that the outcomes in NOD mice can be instructive for some human patients but not all. The overwhelming number of studies in NOD mice and the few viable therapeutics translated to the clinic offer the following lessons: (i) early prevention is simple in NOD mice (more than 65% of studies began some form of therapy between 4–6 weeks); (ii) treating new-onset diabetic NOD mice is difficult; (iii) dosage of the agent, especially putative autoantigen peptide formulation may make or break the success; (iv) most studies in NOD mice cease the monitoring at less than 40 weeks of age; (v) nonspecific microbes can influence the outcome in NOD mice. Considerable effort and logistics have been deployed in the past two decades to use insulin administration as a prophylactic intervention [87], and more recently, to employ an anti-CD3 monoclonal antibody in new-onset human disease [88–90]. The optimism and enthusiasm in both these high-profile multicenter trials was supported by data on the resoundingly successful prevention and “reversal of disease” in NOD mice [91–93]. Nevertheless, insulin administration has yet to demonstrate any successful preventive outcome in humans [87, 94–97], and very recent phase III trials of one embodiment of the anti-CD3 antibody failed to reach the primary end-point (http://www.rttnews.com/Content/BreakingNews.aspx?Id=1451366&SM=1&SimRec=1 and http://www.bizjournals.com/washington/quick_news/2010/10/macrogenics-lilly-abandon-diabetes-drug.html). While “more of the same” autoantigen interventions can be justified by the apparent absence of adverse events noted in the insulin trials (and more recent GAD- and HSP-derived peptide studies), the choice of “more of the same” approaches that involve immunodepletion (chemical or biologic agent-mediated), even if supported by data from NOD mice and BB rats, has to be tempered by unsupportive data in advanced trials as well as borderline outcomes in early-phase trials where heavy statistical treatment might be used to demonstrate efficacy, even if minor. Nevertheless, in the absence of any other animal model, the NOD mouse and the BB rat will continue to be the proving grounds of preclinical efficacy.

## 5. Translation of Successful Preclinical Studies into the Clinic: The Outcomes and the Potential in Humans

The cyclosporin A and the steroid-azathioprine trials proved that established T1DM was reversible [98–100] and, more importantly, that a reserve of beta cell mass was able to restore normoglycemia contingent on suppression of inflammation and autoimmunity [101, 102]. Nevertheless, once cyclosporin A administration was suspended/terminated, the disease reappeared with a vigor no different than that during pretreatment. From a historical perspective, the first successful immunomodulation-based reversal of T1DM was achieved by lymphocyte-specific serum in BB rats [103]. It would be 20 years later that this approach would be attempted in humans [104], although the significant side effects precluded the justification to further explore this approach. Since then, a number of preclinical studies, mainly in NOD mice, have been clinically-translated.

In 1985, Eisenbarth and colleagues reported that steroid-supplemented antithymocyte globulin administration into new-onset T1DM patients reduced insulin requirements. However, this approach was abandoned due to the thrombocytopenia that the procedure was generating [105]. Since then, antithymocyte globulin has been reconsidered and, in new-onset patients, carefully adjusted dosing slowed C-peptide decline [106]. Antithymocyte globulin binds to the CD3 complex on T cells as one of its many targets and causes significantly greater T-cell depletion. Thus, its justification is hampered by significantly greater adverse events and immunosuppression compared to anti-CD3 antibodies.

The initial excitement generated over insulin administration was tempered by the outcome of a major clinical study which showed few, if any, beneficial outcomes [87]. More recent clinical studies using monoclonal antibodies targeting CD3 and CD20 have also been disappointing despite the convincing and robust successes in preclinical studies [65, 89] (http://www.rttnews.com/Content/BreakingNews.aspx?Id=1451366&SM=1&SimRec=1 and http://www.bizjournals.com/washington/quick_news/2010/10/macrogenics-lilly-abandon-diabetes-drug.html).

Long before confirmation in genetic models, insulin and proinsulin were strong contenders as the T1DM-initiating autoantigens [107–114]. As autoantigens, many proposed that the exposure of the T1DM immune system to exogenous insulin in a manner that could modulate immunity towards insulin-specific tolerance could prevent disease in prediabetic states and perhaps delay the progression to overt clinical hyperglycemia in more advanced, but subclinical, states [115–118]. Persistent oral insulin treatment of NOD mice delayed T1DM onset and reduced disease incidence in NOD mice [119]. The addition of adjuvants provided a practical benefit in reducing the amount of exogenous insulin required to achieve the beneficial effects [120]. Largely based on the data supporting the view that mucosal delivery of soluble peptides promotes tolerance to them, a number of efforts targeted, in addition to oral, intranasal insulin or insulin-derived peptide, aerosolisation into prediabetic NOD mice. In these studies, diabetes was significantly delayed [71, 117, 121, 122]. These very promising studies with what essentially was a simple intervention led to the DPT-1 multicenter trial which determined the efficacy of oral insulin in first- and second-degree relatives of T1DM patients deemed to fall into high-risk status based on metabolic, immune, and genetic evidence [73, 123]. Other than a possible benefit in individuals with the highest autoantibody titers, the DPT-1 study failed to delay or prevent T1DM. A similarly disappointing outcome resulted in another prevention trial where insulin was administered intranasally Näntö-Salonen et al, [124]. However, a very small subgroup of autoantibody-positive patients was identified in which some effect was shown. The general view of nonpharmacologic insulin therapy is that, if beneficial, it probably will be restricted to very well-defined and characterised subpopulations of patients. It is unclear what can distinguish these patients from a general at-risk population. 

A number of other trials were initiated since then without any significant benefits. The IMDIAB trial showed that oral insulin provided no benefit over placebo after a one-year followup in terms of mean C-peptide secretion and insulin requirements. The ORALE trial, comparing low- with high-dose oral insulin (2.5 mg/day versus 7.5 mg/day), after one year could not discern any benefit in decelerating the loss of physiologic beta cell function [125]. It was recently shown that combined intranasal insulin with CD3 antibody was able to reverse new-onset T1DM in NOD mice, although the effect was quite likely due to the CD3 antibody and the characteristics of the NOD mouse cohorts and not due to the insulin [126]. 

The most recent attempts at using insulin to improve functional beta cell mass involve intramuscular injection of human insulin B chain in incomplete Freund's adjuvant as well as subcutaneous injection of a DNA plasmid vector encoding proinsulin see [127] and http://www.bayhilltherapeutics.com/. The outcomes of these proposals rest on the publication of phase II studies which are currently at various stages of implementation.

As strong an argument for GAD can be made in the etiopathogenesis of T1DM as for insulin. The 65 KDa isoform of GAD has been demonstrated to be a target of early-insulitic T cells *in vitro* and *in vivo* [128, 129]. Administration of GAD into very young NOD mice suppresses anti-GAD T-cell reactivity as well as disease onset [129, 130]. GAD is equally effective when administered into older NOD mice [130]. The relevance of GAD to human T1DM relies on the presence of GAD autoantibodies in prediabetic humans and is one of three reliable markers of susceptibility [131]. 

Two clinical trials have used human GAD65 with alum as adjuvant to determine improvement of physiologic beta cell function. One was conducted in LADA patients [132] and determined that this formulation increased fasting and stimulated C-peptide at 24 weeks compared to baseline, a benefit that was associated with an increase of CD4+ CD25+ Tregs. The other study considered the effects of GAD65 in alum in recent-onset T1DM individuals (10–18 years of age) on the rate of decline of stimulated C-peptide [133]. The study revealed a slower rate of decline of stimulated C-peptide in GAD-treated diabetics compared to the placebo group. 

Early studies showed that intrathymic administration of Hsp60-derived peptides was prophylactic in NOD mice even though Hsp60 was not considered a bona fide autoantigen involved in T1DM initiation [134]. More experiments in NOD mice revealed that the p277 peptide of Hsp60 was quite effective in suppressing disease progression [135]. These data compelled the development of a human equivalent of p277 (DiaPep277; a 24 amino acid synthetic peptide derived from the C terminus of human hsp60). 

DiaPep277 has been evaluated in phase II studies [136], and data demonstrate some degree of preservation of stimulated C-peptide secretion.

Since then, three other studies have been conducted in new-onset T1DM patients [137–139]. In two of these studies, adult patients were the study population, and these recipients exhibited better, yet limited, C-peptide production than placebo-treated individuals. In contrast, DiaPep277 did not offer any objective benefits in young new-onset patients [137].

T-cell depletion targeting the CD3 complex with the OKT3 monoclonal antibody was successfully employed about two decades ago [91]. Administration of the antibody into diabetic NOD mice resulted in complete remission of disease [91, 92]. One caveat of this particular antibody was that, under some situations, it could activate T cells and therefore it was not considered suitable for human use. Soon thereafter, FcR nonbinding embodiments were manufactured which exhibited either IgG1 Fc chain or which eliminated glycosylation sites of the original OKT3 clone. These variants were found to be less activating than OKT3 [140, 141]. Herold and colleagues initially demonstrated that a short-term administration of the nonglycosylated variant into new-onset T1DM patients suppressed the loss of beta cell function as measured indirectly by surrogate physiologic markers [90, 142]. Some patients maintained improved physiologic markers of beta cell function for as much as two years following the treatment in the absence of systemic immunosuppression. A second round of anti-CD3 antibody injections was postulated as necessary to maintain the beneficial outcome. However, additional injections of the antibody were shown to confer more serious, previously undocumented clinical complications. 

T1DM is unquestionably a T-cell-mediated disease; however, other cell populations have been implicated in the onset, and early progression, of the disease, including B-lymphocytes. Accumulated findings in NOD mice demonstrated that B-cell depletion could be beneficial [143–145], even some studies suggested the contrary [146] where adoptive transfer of T1DM into NOD-SCID mice was possible in the absence of B cells and antibodies. B cells can also participate in disease exacerbation by promoting epitope spreading as shown by Tian and colleagues [147]. Indeed, capture of autoantigens such as GAD65 by B-cell surface immunoglobulin (Ig) is followed by processing and presentation of T-cell determinants by B cells, a step that is crucial for activation of autoreactive T cells and induction of diabetes [148–150]. B-cell-mediated processing of self-Ags may contribute to the generation of an inflammatory microenvironment in the pancreas, which is critical for overcoming the regulatory barrier(s) to initiation of diabetes in NOD mice [151]. Antigen-specific B cells and their antibodies are essential in catalyzing determinant-spreading reaction *via* (a) generation of novel previously cryptic epitopes through altered antigen processing or (b) facilitation of T-cell activation through generation of ligands with higher affinity for TCR and delivery of costimulatory signals. Ag processing through surface receptor-mediated internalization, for example, Fc receptors and soluble Ig, is different from that occurring through pinocytosis and phagocytosis [152].

Pescovitz and colleagues demonstrated a small but statistically significant improvement in physiologic markers of beta cell function in new-onset patients [65]. Nevertheless, the improvement was transient as placebo and rituximab-treated patients eventually (by two years) exhibited an identical decline in beta cell function. One possible mechanism could involve the restriction of epitope spreading by B cells after rituximab-mediated deletion, or the repopulation by B-cells that process and present epitopes that compete with diabetes-relevant epitopes. 

Autoreactive T cells have been frequently associated with the production of TH1-type proinflammatory cytokines. It is reasonable, therefore, to use cytokine blockade as a unique or part of a combination therapeutic strategy. IL-1beta, TNFalpha, and type 1 interferons have been historically the first to exhibit direct beta cell cytotoxicity [153–155]. 

Recently, administration of etanercept, a soluble TNFalpha receptor, in an early-phase efficacy trial resulted in a reduction in the loss of C-peptide in T1DM patients [156]. It is important to note, however, that TNFalpha blockade may have unwanted outcomes; TNFalpha can prevent diabetes in older NOD mice even when splenocytes from diabetic NOD mice are adoptively transferred [157]. Furthermore, TNFalpha blockade may require careful consideration of the age of the individuals being treated since blockade in younger NOD mice prevented disease, while in older NOD mice it accelerated T1DM. 

Even though IL-1beta is now known to exert early-onset impairment in beta cell function and mediates early-phase recruitment of immune cells into islets [158–161], it is unclear if IL-1beta blockade will have any beneficial outcome in prevention of disease or reversal of new-onset disease. In certain populations characterised by metabolic diabetes (LADA, type 2 diabetes), recent trials with the IL-1 receptor antagonist protein (IRAP, Anakinra) improved glucose control [162], and mechanistically it appears that this benefit is achieved by blockade of IL-1beta impairment of immune cell activity (i.e., inflammation) and blockade of IL-1beta effects on beta cell impairment. It is unclear if administration of the IL-1 receptor antagonist protein (Anakinra) will be of any benefit in new-onset cases, especially as the autoimmunity in new-onset disease is largely IL-1beta independent.

## 6. Unconventional Therapies That Might Be Clinically Justifiable

An unexpected recent discovery revolves around the realisation that alpha 1 antitrypsin (A1AT) may possess direct anti-inflammatory and perhaps tolerogenic effects, although they are very likely nonantigen specific. A1AT is a serpin whose administration into new onset T1DM NOD mice reversed disease [163]. In addition to its apparent anti-inflammatory effects on insulitis, it promoted regeneration of beta cells and, very likely in an indirect manner, it improved insulin sensitivity. As unclear as the mechanism of action of A1AT may be, equally unclear is the mechanism of another unconventional T1DM treatment using the antileukemia drug, Gleevec [164].

## 7. Combination Therapies

Recent discussions have focused on combining an immunomodulator successful in preventing/reversing T1DM in preclinical studies and human trials with autoantigen(s) and/or biologic agents that on their own improve the function and/or the mass of residual beta cells. These discussions have also proposed combining two or more immunomodulators. Examples include combining the anti-CD3 antibody with beta-cell-protective and function-enhancing GLP-1 agonists [165, 166], IL-1-neutralising antibodies with the anti-CD3 antibody (http://www.diabetestrialnet.org/studies/index.htm), and anti-CD3 antibodies with traditional pharmacologic immunosuppressives like rapamycin (http://www.diabetestrialnet.org/studies/index.htm).

The problem with these approaches is that the non- anti-CD3 antibody of the combination, on its own had either no effect on prevention or reversal or is not part of any known mechanism that can suppress autoimmunity. Additionally, combining agents where each alone confers obvious side effects could imperil the patient in an additive or multiplicative manner. Last, any benefits a T-cell-depleting antibody may confer on preferential expansion of regulatory T cells could be negated by the effect of pharmacologic inhibitors on Treg function and differentiation. It is our view that combining biologic agents in the absence of solid preclinical data is premature and potentially harmful. It is also unclear, even in the absence of additive adverse events, what improvement addition of another immunomodulator will have on the benefits of the CD3 antibody. 

## 8. Patient-Specific Cell Therapy as a Viable Approach

In 2007, Voltarelli and colleagues first reported that autologous nonmyeloablative hematopoietic stem cell transplantation preceded by cyclophosphamide, granulocyte-colony stimulating factor (G-CSF) stem cell mobilization, and antithymocyte globulin rendered 14/15 new-onset T1DM patients insulin nonrequiring for an average of 16 months [167, 168]. It is unclear if such an approach will be approved elsewhere, indeed in children, and the significant toxicity in all patients calls into question whether this approach is justified for an achievement of 16-month insulin-free state. Nevertheless, hematopoietic stem cell transplantation is clinically feasible and offers one means of either repopulating a depleted immune system with a greater contingent of suppressive immune cells compared to pretreatment or establishing tolerance, even transiently. However, existing autoreactive immune cells will still exist and safe approaches to suppress them must still be employed. 

Cell therapy, especially with dendritic cells, is a clinical reality that has also recently been accepted by health providers (http://www.provenge.com/). Overwhelming studies indicate that downregulated costimulation capacity in DC can subserve a tolerogenic therapeutic outcome [169–175]. Costimulation-impaired (or downregulated), functionally-immature DC administration achieves long term and stable allograft survival in a variety of mouse and rat models and prevents a number of autoimmune diseases [176–183]. Mechanistically, functionally immature DCs. act by inducing anergy either via direct cell contact and/or cytokines [172, 184, 185] or, as described more recently, by upregulating the number and function of immune cell subsets, especially the regulatory populations which include Foxp3+ CD25+ CD4+ T cells and a class of CD8+ immunosuppressive T cells [186–194]. 

Table 1 lists the models where immature DC administration has proven successful. In addition to anergy, Foxp3+ CD25+ CD4+ T-cell numbers have been observed to be increased by the exogenous supply of tolerogenic DC [195–199]. Immature or semimature DC can induce the differentiation of naturally-occurring (thymic) T regulatory cells (CD4+ CD25+) as well as IL-10-producing CD4+ T cells *in vitro* and *in vivo* [195, 198]. Overexpression of the Jagged-1 gene in DC results in the augmentation of antigen-specific TGFbeta-producing CD4+ CD25+ CD4+ T-cells [200]. Functionally immature DCs convert naive T cells into IL-10-secreting cells *in vitro,* and antigen-pulsed DCs injected subcutaneously into humans augment the number of IL-10-producing CD8+ T cells and trigger a reduction in IFNgamma+ T cells [201, 202]. Despite the abundance of data in support of immature DCs as functional inducers of regulatory immune cells, other investigators have discovered that the state of maturity may not be relevant for the induction of immunosuppressive endogenous T cells. For example, similar to the studies by Morel and colleagues [2], mature DCs were shown to expand CD4+ CD25+ CD4+ Foxp3+ T cells which were functionally suppressive and capable of preventing diabetes in the NOD mouse [203–205]. Whether this reflects a restimulation of existing CD4+ CD25+ T cells or their expansion is unknown. We have shown that DCs maintained in a functionally immature state following *in vitro* treatment with NF-kappaB decoys and antisense oligodeoxyribonucleotides (AS-ODNs) to the CD40, CD80, and CD86 costimulatory molecules are diabetes preventive in the NOD mouse [6, 8, 206] and may involve short-range IL-7 signaling, quite likely inside the pancreatic lymph node of NOD mice, to augment the number of CD4+ CD25+ Treg via suppression of apoptosis of a preexisting pool [206]. Multiple injections of AS-ODN-treated DCs in NOD mice maintained the animals as diabetes free without affecting the overall T-cell activity against alloantigens. Furthermore, repeated administrations of co-stimulation-deficient DC reverses hyperglycemia in new-onset diabetes NOD mice. The promising results from this study, together with the low risk of the procedure, helped in gaining approval by the Food and Drug Administration (FDA) office for a phase I clinical trial which has recently ended with very interesting outcomes, the most important being the complete absence of any adverse events. Adult (18 years or older) volunteers with a documented evidence of insulin-requiring T1D of at least 5 years duration were enrolled. Leukocytes of the patients were obtained by apheresis, and DCs were generated *in vitro* and engineered in GMP facilities with the addition of AS-ODN. These DCs expressed low levels of CD80, CD86, and CD40 and were then injected into the patient by intradermal/subcutaneous administration at an anatomical site proximal to the pancreas. The choice of this anatomic region for DC delivery was based on the location of the lymphatic conduits (microvessels) that drain the injection site and favor migration to the pancreatic and para-aortic lymph nodes. We conjecture, based on previous rodent studies, that once inside the pancreatic lymph nodes, the injected DCs will acquire molecules that have drained from the pancreas. These molecules can be acquired by passive drainage or can be transferred to the exogenously supplied DC by antigen-presenting cells coming into the pancreatic lymph nodes from apoptotic islets. Therefore, either by direct uptake of “naked” molecules or antigen transfer by migratory antigen-presenting cells, the exogenously administered DC will acquire the antigen specificity. Alternatively, our administered DC could be recruited from the lymph nodes draining the injection site to the apoptotic islets where they could indirectly deliver an anergizing signal to the T cells they encounter. They can also induce regulatory immune cells. The recent concept of contrasuppression, or alternatively, aggregational suppression (network of intercommunicating DC : Treg) could be operative in our system [207–211]. The vicious cycle that promotes the T cell-mediated anti-beta-cell-antigen-spreading phenomenon will be interrupted this way, enabling the recovery of the physiologic endocrine function of the gland with time. The abrogation of the autoimmune diabetogenic insult should be sufficient to promote rescue or regeneration of the insulin-producing beta cells in the host endocrine pancreas, even after the onset of the disease. Currently, there are no methods to directly test this mechanism *in vivo* in humans; however, mouse and nonhuman primate models offer a number of opportunities. Table 1 lists the many methods to generate tolerogenic DCs. Some methods converge upon identical cellular and molecular pathways (augmentation of Treg numbers, NF-kappaB inhibition, costimulation blockade). Others are not so obvious. A cautionary detail needs insertion herein: it is important to understand that transplantation immunobiology may not be identical to autoimmunity and vice versa. Indeed, there are numerous instances where a therapeutic regimen achieving long-term allograft survival has failed to abrogate autoimmunity [212–219]. Therefore, DCs that are able to suppress donor-specific antigen alloreactivity may not evoke the appropriate regulatory mechanisms capable of controlling and reversing autoimmunity. Also, although different DC products may exhibit similar cell surface phenotypes and nuclear proteome signatures, by no means can this be a predictor of mechanism of action. Cell viability has rarely been examined in these DC products, and not all potential mechanisms of action were considered. It is possible that all listed DC embodiments may intersect at immune regulatory cell levels, but this remains to be determined. The site of action of many DC products, exogenously administered, is currently unknown. While it is generally believed that the DC activity will occur inside the pancreatic lymph nodes, there are equally likely possibilities that the activity of immune tolerance may in fact occur extralymphatically and around the islets, or inside many lymph nodes, anatomically distant from the pancreas. The study by Ludewig and colleagues demonstrating a nascent peri-islet lymphoid ultrastructure during diabetes onset is very interesting and instructive and awaits the discovery of a mechanism [220]. 

Table 1 highlights the studies where animals were treated with tolerogenic DCs pulsed with putative autoantigens. Although the ongoing insulitis drives migration of exogenously administered DCs into the islets, where they acquire the antigen specificity to provide beta cell antigens (from apoptotic/necrotic beta cells) to pancreatic lymph-node-resident regulatory immune cells like Foxp3+ CD25+ CD4+ T cells, it is currently unknown if *ex vivo* pulsing with putative autoantigen(s) could stabilize class I MHC to yield more specific Treg *in vivo*. Our data, however, indicate that the supply of putative autoantigens may not be necessary given the endogenous supply acquired by migratory exogenous DC inside the insulitic lesion. Fathman and colleagues have shown that exogenous DC administration results in preferential accumulation of the DC inside the pancreatic lymph nodes and the omentum [3, 221, 222]. The critical factor in any tissue-targeted approach will be to maintain the DCs in a functionally immature state during and after their accumulation inside that tissue [6, 8, 178, 180, 206]. 

That our CD40-, CD80-, and CD86-impaired DCs exhibited exemplary safety motivates us to proceed with phase II efficacy trials in new-onset T1DM patients with the objective of decelerating the rate of decline of residual beta cell mass, improving glucose profiles, and perhaps reducting insulin requirements. We anticipate beginning such a trial in the next 12–18 months. 

T regulatory cells (Tregs) are another potential cell population that can be used for suppressing the autoimmunity of T1DM. A variety of methods have been employed to expand natural Tregs and to differentiate induced Tregs from peripheral blood progenitors. These cells have been shown to control ongoing autoimmunity and promote diabetes reversal in NOD mice [5, 223–225]. It is unclear whether antigen specificity is relevant in this approach because both antigen-specific and nonspecific expanded Tregs are equally efficacious in suppressing T1DM. Furthermore, Tregs recognising one antigen can have a broad effect on suppression effectors reactive to other antigens [5, 205]. Clinical protocols for T-cell administration into humans exist and patient-specific polyclonal Tregs can, under current conditions, be employed in at least phase I studies. 

A particularly interesting discovery very recently indicating that aryl-hydrocarbon receptor-activated CD4+ T cells preferentially differentiate into stably suppressive Foxp3 Tregs, offers therapeutic opportunities that can be exploited by oral route of drug administration [226, 227]. If safe, orally stable aryl hydrocarbon receptor agonists are developed, one could envisage a situation where mucosal CD4+ T-cells could be induced to differentiate into stably suppressive Foxp3+ Tregs. This could be achieved with chemicals or small molecules alone or formulated into nanodelivery particles. 

Biopolymers offer a unique platform that can carry antibodies, small molecules, chemical drugs, short sequences of DNA/RNA, and even plasmid vectors [228–231]. Nanoparticles and microspheres in particular can be exploited to deliver immunomodulators on their surface, or inside the polymer matrix. Two recent approaches demonstrate that T1DM can be reversed by administration of microparticles carrying immunomodulatory molecules. We have shown that a microsphere formulation comprised of PEG, polyvinyl pyrrolidone (PVP), and poly-L-lysine-hydrobromide and short antisense oligonucleotides (AS-ODNs) targeting the primary transcripts of the CD40, CD80, and CD86 costimulatory genes effectively prevents and reverses type 1 diabetes in the NOD mouse model [232]. Although the mechanism is not yet fully clear, we propose that the microspheres are taken up following subcutaneous injection (close to an abdominal anatomic site drained in part by the pancreatic lymph nodes) by migratory dendritic cells which then accumulate inside the pancreatic lymph nodes. These dendritic cells exhibit downregulated CD40, CD86, and CD80 costimulation (as a consequence of the effects of the antisense), effectively being turned into tolerogenic dendritic cells. In the pancreatic lymph nodes, these dendritic cells could promote increased regulatory T-cell prevalence which, in an antigen-specific manner, could suppress the activation and overall function of autoreactive T cells. Santamaria and colleagues prevented and reversed new-onset disease using nanoparticle-bound autoantigen-derived peptides complexed to MHC proteins. Mechanistically, these investigators showed that the peptide-MHC conjugate was recognised by low peptide-avid autoreactive CD8+ T cells that differentiated into regulatory CD8+ T cells specific for those autoantigen-derived peptides. Furthermore, the use of nanoparticle-conjugated human-relevant peptide-HLA conjugates achieved identical outcomes in human HLA-transgenic diabetic mice [233]. Microparticles offer a unique delivery platform that ranges from injectable to inhalable to topical.

## 9. Conclusion and Perspectives

Table 2 lists past and current immunomodulatory clinical trials and their status. Most have failed to meet their primary end-point. The reasons suggested for the disappointments using autoantigens or derivative peptides can be summarised as follows: (i) the administration route and dosage were not optimal; (ii) peptides may not behave identically to intact proteins and may not promote tolerogenic antigen presentation; (iii) regulatory immune cell responses to peptides are different from protein-derived peptide antigens; (iv) masking critical epitopes *in vivo*; (v) timing of administration and the stage of disease when the intact autoantigens or autoantigen-derived peptides were administered. It is well accepted that advanced-stage disease including clinically T1DM is associated with epitope spreading, so that even if tolerance was achievable to one autoantigen, a number of others that have since appeared would still aggravate autoimmunity.

Reports from recently completed phase III trials using one embodiment of the humanised CD3 antibody were not encouraging. The underlying reasons for why the primary end-points were not achieved, even previous studies were demonstrated to be successful, yet limited in time, are not at all clear at present but could involve one or more of the following: (i) variation among the T1DM patients in actual residual beta cell mass, in that the outcome reflects the enrollment of individuals with a more aggressive autoimmunity and hence poorer residual beta cell mass and (ii) the possibility that the time of administration varied between this phase III and previous trials; it is well known that administration of immunomodulatory agents during different times of the day as well as during different points of biologic cycles can profoundly affect the immune response [234–237]. 

In addition to the anti-CD3 antibody, there are other monoclonal antibodies targeting cytokines as well as fusion proteins interfering with costimulation and serving the role of cytokine decoys. There again, the question is, do these agents represent a promise of a huge leap, an incremental advance, or more of the same? The question is indeed speculative as trials involving some of these agents are ongoing or have not yet started. However, the antibody-based trials are instructive. First, these biologic agents confer variable adverse events. Despite the arguments that these agents can represent antigen-specific approaches, they do not. At best, they promote the homeostatic expansion of new immune cell repertoires that, at various stages of autoimmunity, can act beneficially or can aggravate disease. Recent data indicate that antithymocyte globulin depletion of T cells promotes the homeostatic expansion of T cells with a temporally favorable expansion of naturally occurring (thymic) Foxp3+ Tregs [238]. This observation can guide a better approach to promoting homeostatic expansion of Tregs without considerable T-cell depletion and may be instructive for therapies like the anti-CD3 antibody. This phenomenon also appears possible following B-cell depletion with rituximab (i.e., homeostatic expansion of naive B-cells with favorable expansion of B cells with possible regulatory activity [239]). Thus, if consideration is given to understand the timing of repopulation of the periphery with regulatory cells following T-cell or B-cell depletion, more of the same could yield huge leaps in terms of therapeutic success in new onset T1DM. 

Another consideration that has been discussed but seldom implemented experimentally is the route of administration of autoantigens and derivative peptides with or without adjuvants or immunomodulators/anti-inflammatory agents. Accumulating evidence suggests that the pancreatic lymph nodes are the major, if not the most important, site of autoimmunity triggering and progression [240–243]. Indeed, regulatory immune cells have been identified to expand and to differentiate from precursors inside draining lymph nodes of tissues that are targets of autoimmunity [207, 244–247]. These observations should be instructive in how to approach ongoing autoimmunity with the aim of inducing antigen-specific suppression. As most, if not all, of pancreatic beta cell antigens are concentrated inside the pancreatic lymph nodes by fluid dynamic flow or are brought inside the pancreatic lymph nodes by antigen-presenting cells, they would drive the acquisition of antigen specificity by immune cells modulated inside the same lymph nodes even by an antigen-nonspecific approach. For example, one could administer costimulation-blocking agents in a manner where they accumulate preferentially inside the pancreatic lymph nodes and allow the bulk flow, or the cross-presentation of beta-cell-derived antigens by antigen-presenting cells that have traveled along islet-draining lymphatics, to direct the antigen specificity in conferring antigen-specific immune hyporesponsiveness that is known to occur in response to costimulation blockade.

With the disappointing outcomes of the DPT-1 and related insulin-based trials as well as the failure of industry-sponsored phase III trials with one embodiment of the anti-CD3 antibody, one could reasonably question the value in pursuing further immunodepletion interventions as well as autoantigen-alone trials. There is merit in the argument that the lack of biomarkers to more precisely measure residual beta cell mass that is functional imperils these studies. There is also merit in the arguments that the time of administration of the agents in the phase of the disease, even in new-onset-cases, is critical and needs a comprehensive evaluation. We argue that more of the same cannot go on without such biomarkers and a better understanding of the balance of immunoregulatory cells between the time of clinical onset and 6–12 months from this time in T1DM patients. The variability in immunoregulatory cell numbers and their function has to be securely identified in large populations. In the meantime, many of the resources of research support have been skewed heavily in these approaches with trepidation of supporting cell therapies that are otherwise safe. We propose that cell therapies be seriously considered for at least phase I studies. Furthermore, a new avenue of studies has emerged which attempts to promote accumulation of immunoregulatory cells inside the pancreatic lymph nodes. A better understanding of the pancreatic lymphatics, we believe, will be important to develop these approaches.

It is now evident that the explosion of obesity in the past ten years confounds the diagnosis of T1DM. Metabolic impairments in glucose homeostasis can mask underlying autoimmunity. Furthermore, obesity-related inflammation in individuals genetically at risk for T1DM could accelerate the onset of autoimmunity. A careful examination of the relationship between obesity-related inflammation and autoimmunity progression, we believe, challenges both the “huge leaps” and the “more of the same” approaches in immunomodulation of T1DM. This is relevant not only for obesity, but for LADA as well. In this regard, it is possible that conditioning the obese patient with anti-inflammatory agents will decelerate and permit the detection of underlying autoimmunity. The suppression of inflammation may be beneficial as a front-line conditioning prior to full immunomodulation.

## Figures and Tables

**Table 1:** Models of immunotherapeutic DC administration to prolong graft survival and to treat autoimmunity.

*Autoimmune disease*
Type 1 diabetes
(i) Pancreatic lymph node DC (untreated) [1]
(ii) Mature bone-marrow-derived DC (GM-CSF/IL-4 propagated) or transduced with IL-4 vector [2–4], or TGFbeta [5]
(iii) NF-kappaB oligonucleotide decoy propagated or NF-kappaB inhibition [6, 7]
(iv) Antisense oligonucleotide (CD40-, CD80-, CD86-) propagated [8]
(v) Vitamin D receptor ligands [9, 10]
(vi) Other DC embodiments [11–16]
Thyroiditis
(i) *In vitro* generation with TNFa and supernatant of a GM-CSF-transduced cell line [17, 18]
(ii) GM-CSF generated followed by *in vivo* administration of GM-CSF [19]
Experimental encephalomyelitis (Multiple sclerosis model)
(i) *In vitro* generation with TNFa and supernatant of a GM-CSF-transduced cell line [17, 18]
(ii) VIP and GM-CSF *in vitro* propagation [20, 21]
(iii) TGFbeta and MBP antigen [22]
Myasthenia gravis
(i) RelB knockdown [23]
Arthritis
(i) VIP and GM-CSF *in vitro* propagation [20, 21]
(ii) IL-4- or IL-10-expressing bone-marrow-derived DC and derivative exosome preparations [24–26]
(iii) CD95- (Fas ligand-) expressing bone marrow-derived DC [27, 28]
Gastritis
(i) IL-10- expressing DC [29]
Allergy/asthma
(i) IL-10- overexpressing vector [30]
(ii) Allergen overexpression [31]

*Allotransplantation/xenotransplantation*
Administration of bone-marrow-derived DC from transplant organ donor
(i) DC propagated in low-concentration GM-CSF [32–34]
(ii) DC propagated in GM-CSF, IL-10, TGFb and matured with LPS [35]
(iii) *In vitro* blockade of NF-kappaB by adding aspirin, vitamin D_3_ metabolites/analogues, glucosamine, N-acetyl-cysteine, corticosteroids, cyclosporin A, rapamycin, deoxyspergualin, and mycophenolate mofetil [36–51]
(iv) Gene engineering *in vitro*; DC expressing IL-10, TGFbeta, CTLA-4Ig, indolamine-2,3 dioxygenase, Fas-ligand [52–60]
Administration of transplant-recipient-derived DC prior to transplantation
(i) DC pulsed with class I MHC allopeptide or other alloantigens [61, 62]
(ii) Rapamycin and donor tissue lysate [63]

**Table 2:** Clinical trial history for the immunomodulatory treatment of type I diabetes and current state/outcomes.

Treatment	Clinical phase	Last update	Notes
Anti-CD3	Phase III, canceled	2010	Failed to change patient outcomes, and the phase III study was canceled early [64]
Anti-CD20 (rituximab)	Phase II, completed	2009	Beta cell mass preservation, but no change in C-peptide levels or insulin independence [65]
AS-ODN dendritic cells	Phase I, completed	2011	Treatment safety demonstrated
GAD65 protein (Diamyd)	Phase III, Ongoing	2010	Phase II displayed elevated anti-inflammatory cytokines and Treg cells. Insulin independence was not addressed [66, 67]
HSP60 (DiaPep277)	Phase III, ongoing	2008	Phase II trials display a trend of increased C-peptide levels, anti-inflammatory cytokines, and anti-inflammatory T-helper 2 cells [68]
Insulin APL (NBI-6042)	Phase II, failure	2009	Beta cell mass was unaffected [69, 70]
Insulin (intranasal)	Pilot	2004	Decreased T-cell responsiveness to insulin in patients expressing two to three diabetes-related autoantibodies [71, 72]. Additional clinical trials (phase I–III) are underway
Insulin (oral)	Phase I, failure	2005	Initial trials showed no prevention or delay of type 1 diabetes, but additional trials are underway [73, 74]
